# Antimicrobial Resistance in *Escherichia coli* from Hedgehogs (*Erinaceus europaeus*) Admitted to a Wildlife Rescue Center

**DOI:** 10.3390/ani15152206

**Published:** 2025-07-27

**Authors:** Ilaria Prandi, Alessandro Bellato, Patrizia Nebbia, Onésia Roch-Dupland, Maria Cristina Stella, Elena Passarino, Mitzy Mauthe von Degerfeld, Giuseppe Quaranta, Patrizia Robino

**Affiliations:** 1 Department of Veterinary Sciences, University of Turin, Largo Paolo Braccini, 2, 10095 Grugliasco, TO, Italy; ilaria.prandi@unito.it (I.P.); patrizia.nebbia@unito.it (P.N.); onesia.roch_dupland@edu.unito.it (O.R.-D.); mariacristina.stella@unito.it (M.C.S.); patrizia.robino@unito.it (P.R.); 2 Centro Animali Non Convenzionali, Department of Veterinary Sciences, University of Turin, Largo Paolo Braccini, 2, 10095 Grugliasco, TO, Italy; elena.passarino@edu.unito.it (E.P.); mitzy.mauthe@unito.it (M.M.v.D.); giuseppe.quaranta@unito.it (G.Q.)

**Keywords:** European hedgehogs (*Erinaceus europaeus*), *Escherichia coli*, antimicrobial resistance, ECOFF, wildlife rescue center, hospitalization, minimum inhibitory concentration (MIC)

## Abstract

Among synanthropic species, European hedgehogs are widely distributed throughout Europe. In recent decades, these animals have increasingly adapted to anthropogenic environments, where they find abundant shelter and food resources, along with fewer natural predators. As with other wildlife, it is likely that their coexistence in cities is also affecting their microbiota, promoting the development of antimicrobial resistance (AMR). This study aimed to assess the occurrence and patterns of AMR in commensal enteric *Escherichia coli* isolated from hedgehogs (n = 53) living in anthropogenic environments upon admission to a wildlife rescue center in Turin (Italy). The effects of hospitalization on the prevalence and trends of AMR were also assessed. Our results confirm that hedgehogs can harbor resistant *E. coli* upon admission, in particular against cefazolin (41.5%), ampicillin (37.7%), and enrofloxacin (22.6%). In addition, hospitalization promoted an increase in minimum inhibitory concentration (MIC) values of all antibiotics except imipenem, which led to a significant increase in *E. coli* that was resistant towards doxycycline, enrofloxacin, and trimethoprim-sulfamethoxazole. Admitted hedgehogs were also carriers of extended-spectrum beta-lactamase-producing *E. coli* (5.7%), whose presence increased during hospitalization (to 20.8%). These results highlight the role of hospitalizations longer than five days in the acquisition of AMR and suggest that European hedgehogs can become potential carriers of resistant *E. coli* following hospitalization.

## Simple Summary

Studies on antibiotic resistance levels in wild animals and how this is affected by hospitalization are lacking. The present research aimed to evaluate the presence and patterns of resistance in enteric *E. coli* isolated from hedgehogs living in human environments and brought to a wildlife rescue center for recovery. The impact of hospitalization on antibiotic resistance was also assessed, with hedgehogs tested after a number of days. We found that over half of newly hospitalized animals harbored *E. coli* that was resistant to at least one antimicrobial, with an increase after a hospitalization period. The highest number of resistant strains was observed against antibiotics commonly used in non-conventional species. Our results confirm that hospitalization can also increase antimicrobial resistance in small wild mammals, potentially resulting in them carrying resistant strains or resistance genes after release.

## 1. Introduction

*Escherichia coli* constitutes a major component of the intestinal microbiota in warm-blooded animals [1,2]. Due to its ecological versatility, coupled with its ability to easily acquire resistance genes, it is often used as a bioindicator of acquired antimicrobial resistance (AMR), induced by humans directly through the administration of antibiotics or, indirectly, via environmental transmission [3,4]. Regarding resistance patterns, *E. coli* is becoming increasingly resistant to some critically important antibiotic classes like fluoroquinolones and third-/fourth-generation cephalosporins [4,5]. Resistance to beta-lactam antibiotics is mainly mediated by extended-spectrum beta-lactamase (ESBL)- and AmpC-producing *E. coli* [4]. The spread of ESBLs is of particular concern to public health because they confer resistance to a broad range of beta-lactam antibiotics, including penicillins, third- and fourth-generation cephalosporins, and monobactams, thereby limiting available therapeutic options [6,7].

In recent decades, the destruction of wild habitats, human population growth, and urban expansion have increased contact between humans and wild animals [4,8]. In this context, some species have adapted to urban environments, living in closer proximity to humans [9], leading to more frequent interactions and, consequently, to a greater risk of AMR diffusion [8]. Therefore, it is crucial to study AMR levels in synanthropic species, as well as their mechanisms of acquisition and transmission [10].

Among synanthropic species, European hedgehogs (*Erinaceus europaeus*) are widely distributed across Europe [11]. In recent decades, they have increasingly adapted to urban environments, where they find abundant shelter, ample availability of prey and food sources, and few natural predators [12]. In cities, hedgehogs frequently forage in residential gardens and urban parks, where residents provide them with pet food [13], increasing the likelihood of direct contact. Conversely, in rural areas, exposure to livestock and their manure in farmyards and crop fields [14] increases the likelihood of hedgehogs acquiring resistant bacteria associated with human and livestock activity, thus making them potential indicators of AMR pollution in anthropized environments [15]. Additionally, the possibility of transmitting resistant bacteria to humans who feed them in urban settings cannot be ruled out [15,16].

Recently, wildlife rescue centers (WRCs) have noted an increase in the number of European hedgehogs admitted [17]. Hedgehogs harboring AMR bacteria and requiring medical care may not only fail to respond to antimicrobial therapy but also pose a zoonotic risk to WRC personnel [15,18]. On the other hand, during their stay at WRCs, hedgehogs are exposed to several risk factors that may increase their likelihood of acquiring AMR. These include prolonged contact with humans and exposure to diets and environments that differ from their natural habitats, potentially leading to alterations in gut microbiota [19,20]. Furthermore, it has been documented in humans as well as in various domestic and wild animal species that hospitalization, its duration, and antibiotic therapy can contribute to the spread of AMR [21,22,23]. Indeed, the extensive use of antibiotics in hospital settings favors the survival of resistant strains while inhibiting the growth of susceptible ones [3]. Furthermore, during hospitalization, resistant bacteria can be obtained from the environment, even indirectly, or through contact with other animals [19,24]. Antimicrobial treatments and hospitalization have the potential to alter the composition of gut microbiota for months, with AMR bacteria persisting for extended periods [22,25]. In WRCs, this means that animals are released back into their natural environment with altered microbial composition, one that is rich in AMR bacteria. This could reduce their adaptability to their ecological niche and increase potential conservation risks [19]. Furthermore, this promotes the spread of AMR in the environment, affecting other wild species, livestock, and humans [19,23,26,27]. This may result in the release of hedgehogs into the wild carrying higher levels of AMR than they did upon admission to the WRC, which could further disseminate resistant bacteria in the environment [19,26]. Therefore, in urban areas this can facilitate the direct or indirect acquisition of AMR bacteria by citizens and domestic animals [15].

This study aimed to assess the AMR levels of *E. coli* isolated from European hedgehogs upon admission to a WRC, and how they are affected by hospitalization.

## 2. Materials and Methods

### 2.1. Animals Involved in the Study and Housing Characteristics

The study was conducted from May 2023 to July 2024 on European hedgehogs admitted to the Centro Animali Non Convenzionali (C.A.N.C.) WRC, a section of the Veterinary Teaching Hospital at the University of Turin, Northwestern Italy. All animals were collected in urban and suburban areas in the Turin province by citizens who brought them to the WRC because they considered the hedgehogs in need of medical assistance. None of the hedgehogs were kept as companion animals, in accordance with current Italian legislation (Law 152/1992) prohibiting the domestication of *E. europeaus* [28].

Upon admission, anamnestic data were collected (e.g., age, sex, place of origin, and reason for admission), and a clinical examination was performed. The animals included in the study met the following criteria:

(i) Adult subjects;
(ii) A clinical condition requiring hospitalization but not intensive care, as the aim was to investigate the effect of hospitalization without compromising the health or prognosis of critically ill individuals (e.g., with severe injuries or pathologies);
(iii) Not showing gastrointestinal symptoms;
(iv) An interval of no more than 12 h between their discovery and admission to the center, to minimize potential contamination from the rescuer’s environment;
(v) Being alive and still hospitalized at the time of the second sampling.

Based on these parameters, a total of 121 European hedgehogs were sampled at the moment of admission to the center. When animals presented clinical signs compatible with an infectious disease, an antimicrobial therapy was administered. After clinical examination and sample collection, each animal was housed individually in a cage with metal bars, a plastic floor, and a paper substrate, which was cleaned daily. All hedgehogs were kept in the same room, with cages arranged on different shelves next to each other. During the hospitalization period, individuals of various other wildlife species (both birds and mammals), with or without prescribed antibiotic therapy, were housed in the same room. Hedgehogs were fed a mixture of wet cat food, fruit, and dried insects, administered at sunset, while water was provided ad libitum. Throughout handling and clinical procedures, personnel at the WRC consistently used personal protective equipment (PPE), primarily for their own protection, but also to limit the potential transfer of human-associated microbiota to the animals.

### 2.2. Sample Collection

At admission (T0), as part of the clinical examination, each hedgehog underwent gaseous anesthesia, during which a rectal swab (Copan Transystem™ 108 C.USE, Copan Italia S.p.A., Brescia, Italy), was collected before the administration of any antimicrobial drug (if necessary). A second rectal swab was collected after a median of 10 days (T1) of hospitalization (min–max = 5–15 days), with the exact timing adjusted according to the clinical condition and management needs of each individual. For ethical reasons, the T1 sampling was performed opportunistically to minimize handling and associated stress, which is particularly relevant for wild animals, and to avoid habituation to human presence. For example, if an animal was scheduled for discharge before day 10, the T1 sample was collected at discharge; if a medical procedure requiring anesthesia occurred around day 10, the sample was taken at that time to avoid additional manipulation; similarly, if discharge was planned a few days after day 10, the sample was also collected at that point.

Swabs were transported to the laboratories of the Infectious Disease Unit of the Department of Veterinary Sciences, University of Turin, and stored at 4 ± 2 °C until processing (within 24 h).

### 2.3. Phenotypic Analysis

Each swab was streaked onto a MacConkey 3 agar plate (McC3, Oxoid Ltd., Basingstoke, UK) and aerobically incubated at 37 ± 1 °C for 18–22 h. Pink/red colonies were considered indicative of *E. coli* presence and were identified using Matrix-Assisted Laser Desorption/Ionization—Time of Flight (MALDI-ToF) mass spectrometry (Bruker Daltonics GmbH, Bremen, Germany), following the manufacturer’s instructions. As per the manufacturer’s instructions, a score higher than 1.99 was considered indicative of high-confidence species identification.

After species confirmation, five to ten *E. coli* colonies were pooled from each sample and stored at −80 °C in 0.5 mL of Tryptone Soya Broth (TSB, Thermo Scientific, Waltham, MA, USA) with 15% glycerol (Sigma-Aldrich, Darmstadt, Germany) for further analyses. The collection of multiple *E. coli* colonies per animal aimed to obtain a sample representative of the within-host *E. coli* diversity, as different *E. coli* strains can simultaneously colonize a single individual [29].

To screen for the presence of ESBL-producing *E. coli*, rectal swabs were also pre-enriched in buffered peptone water (BPW) and incubated at 37 ± 1 °C for 18–22 h. After the incubation, a loopful of the culture was transferred onto McC3 agar supplemented with 1 mg/L of cefotaxime (CTX), and the plates were incubated overnight at 37 ± 1 °C for 18–22 h. Up to three *E. coli*-like colonies were identified by MALDI-ToF. To confirm the presence of ESBL-producing *E. coli*, pools including identified *E. coli* colonies were tested with a Cefpodoxime Combination Disc Kit (Oxoid Ltd., Basingstoke, UK), according to the manufacturer’s instructions. After 18–22 h at 37 ± 1 °C, a difference of >5 mm between the two inhibition zone diameters was considered positive for ESBL-producing *E. coli*.

Antimicrobial susceptibility was evaluated by determining the minimum inhibitory concentrations (MICs). Pooled colonies from all samples were tested with COMPGN1F Sensititre plates (Thermo Scientific, Waltham, MA, USA) for their susceptibility to a panel of 19 antibiotics, following the manufacturer’s instructions. The tested antibiotics (EUCAST abbreviation; testing range) were as follows: amikacin (AMI; 4–32 mg/mL), amoxicillin–clavulanic acid 2:1 ratio (AMC; 0.25/0.12–8/4), ampicillin (AMP; 0.25–8), cefazolin (CZO; 1–32), cefovecin (CVE; 0.25–8), cefpodoxime (CPO; 1–8), ceftazidime (CTZ; 4–16), cephalexin (CLE; 1–16), chloramphenicol (CHL; 2–32), doxycycline (DOX; 0.25–8), enrofloxacin (ENR; 0.12–4), gentamicin (GEN; 0.25–8), imipenem (IMI; 1–8), marbofloxacin (MAR; 0.12–4), orbifloxacin (ORB; 1–8), piperacillin–tazobactam constant 4 mg/mL (PIT; 8/4–64/4), pradofloxacin (PRA; 0.25–2), tetracycline (TET; 4–16), trimethoprim–sulfamethoxazole (TRS; 0.5/9.5–4/76).

### 2.4. Data Analysis

Data handling and statistical analysis were performed with R version 4.4.3 [30], using the packages car (version 3.1.3) [31], lme4 (version 1.1.37) [32], lmerTest (version 3.1.3) [33], survival (version 3.5.8) [34], and survminer (version 0.5.0) [35].

#### 2.4.1. Minimum Inhibitory Concentration

For each antimicrobial, the MIC values at T0 and T1 were visually evaluated through bar plots and compared using a Wilcoxon’s signed rank test for paired data. Based on the results observed in Prandi et al. [20], we hypothesized that the MIC would increase after hospitalization. Therefore, we performed a one-tail test considering a significance level of α = 2.5%. Nonetheless, *p*-values were treated as continuous measures of compatibility with the null hypothesis rather than being dichotomized by a fixed threshold.

To determine whether the antimicrobial therapy, the hospitalization, or the duration of the hospitalization were associated with the change observed in MIC, we employed a Generalized Linear Mixed Model. As fixed effects, the model included the sampling (T0 vs. T1), the duration of hospitalization (in days), and a binary variable indicating whether the patient was administered antibiotic therapy, regardless of the administered compound. Also, the model included the patient as random effects (random intercepts).

(1) $$MIC=\beta_{0}+\beta_{1}\times sampling+\beta_{2}\times duration+\beta_{3}\times therapy+u_{i}+\varepsilon$$

where MIC is the logarithmic transformation in base 2 of the MIC; $\beta_{0}$ is the baseline MIC; $\beta_{1-3}$ are the coefficients of sampling, duration of hospitalization and therapy, respectively; $u_{i}$ is the random intercept estimated for each individual; and ε is the random error. We performed forward stepwise selection based on the Akaike Information Criterion (AIC) to determine which set of variables best fit each antimicrobial. We did not apply a fixed ΔAIC threshold (e.g., >2) but assessed model fit balancing goodness-of-fit with model complexity.

The asymmetric 95% confidence intervals (CI) were estimated via profile likelihood, without normality assumptions. Coefficient and CI estimates were converted by entering them as powers of two (i.e., $2^{\beta_{x}}$) so that their effect could be interpreted in relation to MIC.

#### 2.4.2. Antimicrobial Resistance

For European hedgehogs, no specific clinical breakpoints were available. Therefore, the MIC values were interpreted using epidemiological cut-offs (ECOFFs) and tentative ECOFFs (TECOFFs) provided by EUCAST [36]. (T)ECOFFs distinguish microorganisms without (wild-type, MIC ≤ (T)ECOFF) and with phenotypically detectable acquired resistance mechanisms (non-wild-type, MIC > (T)ECOFF) to an antimicrobial agent [37,38].

Based on (T)ECOFF, wild-type (WT) and non-wild-type (nWT) *E. coli* isolates were determined. This evaluation was carried out for all the antimicrobials that had a published (T)ECOFF. For cefovecin, due to the absence of a reported (T)ECOFF, we used the cut-off reported by Stagemann et al. [39]. To the best of the authors’ knowledge, marbofloxacin, orbifloxacin, and pradofloxacin did not have reported cut-offs. Therefore, resistance was not assessed for these antimicrobials.

To enhance the readability of the text, the authors occasionally refer to nWT strains as “resistant,” based on the fact that nWT strains possess at least one phenotypically detectable acquired resistance mechanism.

The proportion of hedgehogs harboring nWT *E. coli* was calculated at T0 and T1 and compared by one-tail Fisher’s exact test with a significance level of α = 2.5%. However, *p*-values were interpreted as continuous measures of compatibility with the null hypothesis, not dichotomized by a fixed threshold. In cases where we observed a difference in nWT proportion, we calculated the odds ratio (OR). The 95% CI of the proportion of nWT was calculated by Wald’s approximation:

(2) $$95\%C.I.=Prop\;\pm \;1.96\;\times \;\sqrt{\frac{Prop\;\times \;(1\;-\;Prop)}{N}}\;$$

where *N* is the number of samples.

Additionally, to investigate the time it takes for the acquisition of a nWT *E. coli* by a patient that was not harboring it at admission, survival analysis was performed. This analysis accounted for censored observations too, i.e., individuals discharged from the WRC prior to acquiring a nWT *E. coli*. The probability of not acquiring a nWT *E. coli* during hospitalization was modelled with a Kaplan–Meier curve:

(3) $$S\left(t_{j}\right)=S(t_{j-1})\;\times \;(1-\frac{d_{j}}{n_{j}})$$

where $S(t_{j-1})$ is the probability of not harboring a nWT *E. coli* at time $t_{j-1}$; $n_{j}$ is the number of individuals not harboring a nWT *E. coli* just before $t_{j}$; and $d_{j}$ is the number of events at $t_{j}$. The function assumed $t_{0}=0$ and $S\left(0\right)=1$. The hazard probability was obtained as follows:

(4) $$H\left(t\right)=-ln(S\left(t\right))\;$$

The patients were divided based on the administration of antimicrobial therapy. To compare the hazard between treated and untreated subjects, we employed the non-parametric log-rank test, which does not require assumptions about the survival distribution.

#### 2.4.3. Extended-Spectrum Beta-Lactamase-Producing *E. coli*

The prevalence of hedgehogs harboring ESBL-producing *E. coli* was calculated at T0 and T1 and compared by Fisher’s exact test. Where we observed a difference in the proportion of ESBL-producing isolates between T0 and T1, the OR was calculated.

In addition, survival analysis was performed to investigate the factors affecting the acquisition of an ESBL+ *E. coli* by a patient that had not been harboring it at admission. Since this event may not have occurred for some individuals within the study period, censored observations were handled by survival analysis. The probability of acquisition of an ESBL-producing *E. coli* was modelled with a Kaplan–Meier curve (2), and the hazard probability was obtained (3). The patients were divided based on the administration of antimicrobial therapy. Treated and untreated animals were compared using the non-parametric log-rank test.

## 3. Results

During the study period, a total of 121 animals were sampled. Of these, 61 hedgehogs were either released or died before the second sampling, while the remaining 60 animals were sampled at both time points (T0 and T1) and, thus, enrolled in the study. The median stay of enrolled animals was 10 days (minimum 5 days, maximum 15 days).

Out of 60 animals, 26.1% (n = 16) did not show any clinical symptoms, 28.3% (n = 17) were lethargic, 10.9% (n = 7) were visibly infested with ectoparasites, 8.7% (n = 5) showed respiratory distress, 6.5% (n =4) had upper respiratory tract symptoms, and 6.5% (n = 4) had wounds. Other reasons for admission included abscesses, phlegmon, trauma, and periodontitis.

Ten out of 60 animals (16.7%) required antimicrobial therapy during hospitalization because they exhibited respiratory symptoms (n = 3), had a contaminated wound (n = 3), or had active infections (n = 4). Nine of them received enrofloxacin (5–10 mg/kg twice daily [bid], via subcutaneous injection [sc], appropriately diluted to minimize its tissue-irritating activity, while one was treated with trimethoprim–sulfamethoxazole (30 mg/kg bid, sc) [40].

### 3.1. Microbiological Analysis

At T0, *E. coli* was not isolated from two out of the 60 animals, resulting in a detection rate of 96.7% (95% CI: 88.5–99.6). After ten days, *E. coli* was detected in 91.7% of the animals (n = 55, 95% CI: 81.6–97.2). In total, 53 animals were positive for *E. coli* at both time points and were tested for antimicrobial susceptibility.

### 3.2. Minimum Inhibitory Concentration

From T0 to T1, the MIC of all antibiotics except imipenem increased. A non-significant and less marked increase was observed for all beta-lactams (AMP, AMC, and PIT). Among cephalosporins, a non-significant increase was witnessed for cephalexin, cefpodoxime, and ceftazidime. Conversely, the MIC of cefazolin and cefovecin significantly increased (*p* = 0.015 and *p* = 0.007, respectively). We estimated a 1.42 µg/mL (95% CI: 1.02–1.99 µg/mL) increase in the MIC of cefazolin from T0 to T1, and 1.27 µg/mL (95% CI: 1.05–1.52 µg/mL) for cefovecin. The antimicrobial therapy was not associated with the MIC increase.

All tested fluoroquinolones registered a significant increase in MIC from T0 to T1, not related to antimicrobial therapy. The steepest increase was observed for enrofloxacin (*p* = 0.003), which rose by 1.75 µg/mL (95% CI: 1.16–2.65 µg/mL), followed by marbofloxacin (*p* = 0.015), which rose by 1.56 µg/mL (95% CI: 1.02–2.33 µg/mL), and pradofloxacin (*p* = 0.017), which increased by 1.39 µg/mL (95% CI: 1.04–1.85 µg/mL) from T0 to T1. The estimated increase in orbifloxacin was 1.33 µg/mL (95% CI: 1.02–1.75 µg/mL; *p* = 0.025).

Also, aminoglycoside MIC significantly increased from T0 to T1 but not in association with antimicrobial therapy. The amikacin MIC was estimated to have increased by 1.17 µg/mL (95% CI: 1.04–1.32 µg/mL; *p* = 0.007). The increment of gentamicin MIC was proportional to the duration of the stay and estimated to be on average 1.03 µg/mL × day^−1^ (CI: 1.01–1.06 µg/mL × day^−1^; *p* = 0.010).

For tetracyclines we observed a significant increase in MIC, too (*p* = 0.017). Tetracycline MIC increased by 1.39 µg/mL (CI: 1.04–1.85 µg/mL) from T0 to T1, not in association with antimicrobial therapy. On the other hand, the doxycycline MIC increase was not only proportional to the duration of the stay, with an average 1.05 µg/mL increment per day of hospitalization (CI: 1.03–1.08 µg/mL × day^−1^; *p* < 0.001), but also associated with antimicrobial therapy. We estimated an additional 1.70 µg/mL increase related to antimicrobial therapy (CI: 1.00–2.82 µg/mL).

Similarly, trimethoprim–sulfamethoxazole MIC increased by an additional 2.11 µg/mL (CI: 1.15–3.85 µg/mL) related to antimicrobial therapy, in addition to the 1.19 µg/mL (CI: 1.00–1.41 µg/mL) increase from T0 to T1 (*p* = 0.044).

Lastly, chloramphenicol MIC significantly increased by 1.37 µg/mL (CI: 1.11–1.69 µg/mL; *p* = 0.002), not in association with antimicrobial therapy.

MIC distributions at T0 and T1 for each tested antibiotic are displayed in Table 1.

### 3.3. Antimicrobial Resistance

Of the 53 hedgehogs carrying *E. coli* at admission, 60.4% (n = 32, 95% CI: 47.2–73.5) carried nWT isolates for at least one antibiotic. At admission, the highest number of nWT isolates was observed for cefazolin (CZO, n = 22, 41.5%, 95% CI: 28.2–54.8), followed by ampicillin (AMP, n = 20, 37.7%, 95% CI: 24.7–50.8), enrofloxacin (ENR, n = 12, 22.6%, 95% CI: 11.4–33.9), and amoxicillin–clavulanic acid (AMC, n = 11, 20.8%, 95% CI: 9.8–31.7). The proportion of nWT for other antibiotics did not exceed 20% (Table 1).

**Figure 1:**
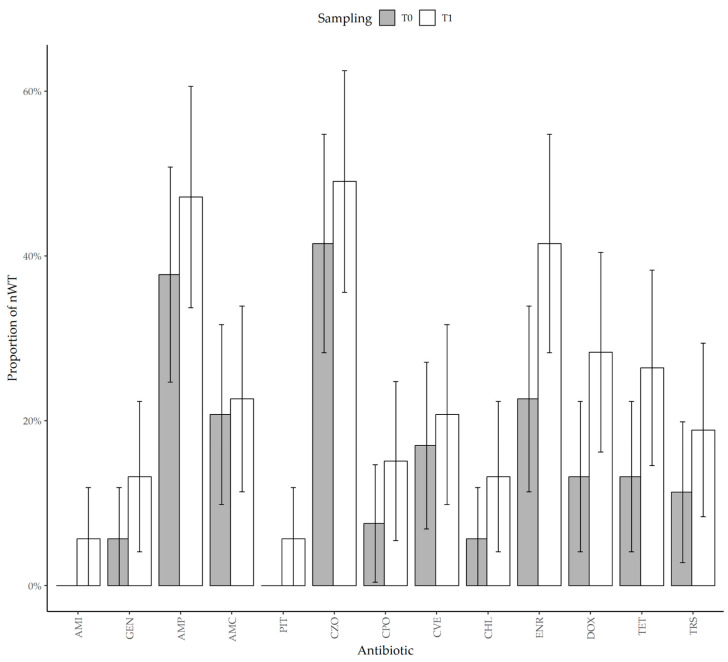
Proportion of non-wild-type *E. coli* isolated at T0 and T1 for each antibiotic tested with an available (T)ECOFF.

Since the (T)ECOFF fell outside the tested range, we had indeterminate results for imipenem, cefalexin, and ceftazidime. For all the other antibiotics, we observed an increase in the nWT proportion (Figure 1).

However, the increase was not significant for beta-lactams (AMP, AMC, PIT), aminoglycosides (AMI, GEN), tetracycline, and chloramphenicol.

Among cephalosporins, we observed a non-significant increase for all antibiotics, but the antimicrobial therapy was associated with a higher proportion of nWT at T1 for cefazolin (*p* = 0.021) and cefovecin (*p* = 0.003). Although the ECOFF for ceftazidime fell outside the tested MIC range, two animals that carried indeterminate isolates at T0 were found to carry nWT isolates at T1.

The proportion of nWT significantly increased for doxycycline from 13.2% at T0 to 28.3% at T1 (*p* = 0.010), for enrofloxacin from 22.6% at T0 to 41.5% at T1 (*p* < 0.001), and for trimethoprim–sulfamethoxazole from 11.3% at T0 to 18.9% at T1 (*p* = 0.044).

**Figure 2:**
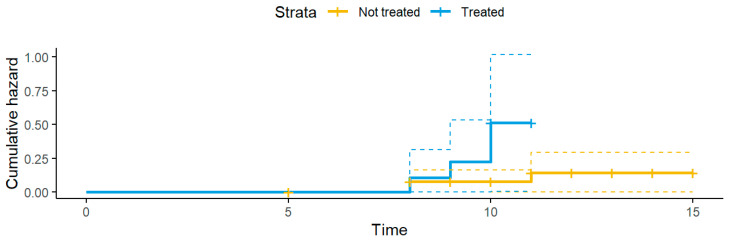
Hazard curves of hedgehogs during hospitalization. The figure represents the increase in the number of treated and non-treated hedgehogs harboring an ESBL-producing *E. coli* during hospitalization. Dashed lines represent 95% confidence intervals for the cumulative hazard of treated (blue) and not treated (yellow) hedgehogs.

At T0, three hedgehogs out of 53 (5.7%) harbored an ESBL-producing *E. coli*. At T1, the proportion of ESBL-producing *E. coli* had significantly increased to 20.8% (n = 11; *p* = 0.021). The increase was significantly associated with antimicrobial therapy (*p* = 0.016) (Figure 2).

## 4. Discussion

Our results underscore the presence of AMR *E. coli* in the intestinal tract of European hedgehogs and show that a period of hospitalization longer than five days at a WRC promotes an increase in MIC values and in AMR levels.

At admission, *E. coli* was detected in 96.7% of the tested hedgehogs. Detection rates of *E. coli* vary widely across different species [41], and in Europe, it has been found in wild animals at rates ranging from 23% [42] to 100% [4], confirming its high prevalence in the gastrointestinal tract of warm-blooded species [2].

WRCs provide a valuable opportunity to study wild animals without interfering in their natural environment and enable the collection of a large number of samples [23]. However, animals admitted to WRCs are often stressed, injured, or ill and in need of medical assistance, factors that can alter the composition of their gastrointestinal microbiota [43]. Consequently, the bacterial populations sampled in WRCs may not fully represent the prevalence and characteristics of healthy wild animals. To minimize this bias, samples were collected immediately upon admission, and animals that had been kept by rescuers for more than 12 h or which presented with critical injuries or severe pathologies were excluded from the study.

Despite not having been previously treated with antibiotics, wild hedgehogs admitted to the WRC carried *E. coli* that exhibited moderate-to-high levels of resistance, particularly to cephalosporins, penicillins, and fluoroquinolones. The most frequent resistances were observed against cefazolin (41.5%), ampicillin (37.7%), enrofloxacin (22.6%), and amoxicillin–clavulanic acid (20.8%). These findings are partially consistent with reports from other countries, where in wild animals, including hedgehogs, from Europe and beyond, the most common resistances in commensal *E. coli* have been reported against penicillins, tetracyclines, and antifolates, with resistance rates reaching up to 87.4% for amoxicillin–clavulanic acid and 68.6% for ampicillin [4,9,18,44]. We detected the highest resistance towards first-generation cephalosporins and resistance towards third-generation cephalosporins ranged between 7.6% and 17.0%. In addition, fluoroquinolone resistance averaged 22.6%. High resistance levels to first-generation cephalosporins have been detected previously in wild animals, with 94.3% cephalothin-resistant *E. coli* detected in wild boars and 84.6% cephalexin-resistance in wild birds in Italy [20,44], and 58% cephalexin-resistant wild species in Costa Rica [23]. Contrary to our results, resistance towards third-generation cephalosporins is low both in wild and farming animals in Europe (0.0–0.9%) [8,41,45]. However, third-generation cephalosporin-resistant *E. coli* have also previously been detected in Costa Rican wild animals (4–7%) [23]. Furthermore, reports on fluoroquinolone resistance range widely, from 0.0–0.9% in wildlife from Germany to 13.7% in Italian wild boars and farming animals (26–42%) [44,46], reflecting potential differences in the distribution of fluoroquinolone resistance in different environments. Both in our and previous studies, no amikacin and carbapenem resistance was detected in commensal *E. coli* isolated from wild animals [10,23], indicating its low-to-absent presence in the urban and peri-urban niches where they live. We detected 5.7% of ESBL-producing *E. coli*, which have also been previously detected in European hedgehogs from Spain with detection rates of up to 13.5% [4,15,47].

However, as no clinical breakpoints are currently available for *E. europaeus*, MIC values were interpreted using available ECOFFs and TECOFFs. These thresholds are appropriate for epidemiological purposes, such as detecting shifts in bacterial susceptibility, but do not allow inference of the likely clinical efficacy of antimicrobial treatment and may limit comparability with studies applying clinical breakpoints or involving other host species.

Variations in the resistance to different antimicrobial classes of wild species all over the world could be attributed to different environments, diets, or grade of contact with anthropogenic activities and domestic or farming animals [8,15,48]. Overall, the studied European hedgehogs and previously reported wild species have been detected to be potential carriers of high levels of AMR bacteria and ESBL-producing *E. coli*. Resistant genes and bacteria can be acquired from the environment, where wastewater, sewage, hospital discharge, manure from livestock treated with antibiotics, and other human activities promote the spread of AMR determinants [8,9,49]. In addition, as insectivores, hedgehogs feed on worms, snails, and other invertebrates that can acquire and transmit AMR bacteria from contaminated soil [50,51].

With sufficient hospitalization time, an increase in MIC values was observed for all antibiotics except imipenem, whose MICs remained below detectable levels at both T0 and T1 (although, for some antibiotics, the values remained below the ECOFF threshold). This increase was proportional to the length of hospital stay for gentamicin, doxycycline, and trimethoprim-sulfamethoxazole. Additionally, a significant rise in non-wild-type (nWT) strains was detected for enrofloxacin, doxycycline, and trimethoprim–sulfamethoxazole. Also, ESBL-producing *E. coli* displayed a significant increase during hospitalization and antimicrobial treatment. Antimicrobial therapy was significantly associated with an increase in nWT strains for cefazolin, cefovecin, doxycycline, and trimethoprim–sulfamethoxazole. This may be due to the direct or indirect exposure of the hedgehogs to antibiotics used in the WRC. In particular, similar to our results, several studies reported an increase in fluoroquinolone resistance, reaching a prevalence of over 60% [19,20,23]. The consistent increase in fluoroquinolone resistance can be explained by the frequent use of these antimicrobials in veterinary practice, especially in WRCs [10,20], where they are often the only antibiotics registered for non-conventional species due to regulatory constraints. Accordingly, enrofloxacin was commonly used in our setting, which likely contributed to the selection pressure observed in the sampled animals. Indeed, our and other studies have highlighted a significant association between antimicrobial administration, especially of fluoroquinolones, and the acquisition of AMR bacteria [20,21,52,53,54]. Antimicrobials suppress susceptible microorganisms that are harbored in the gastrointestinal tract, promoting consequent colonization by resistant mutants, which can already be part of the intestinal microbiota at low concentrations or can be acquired during the hospital stay [21]. Also, the duration of hospitalization has been linked to AMR, with longer hospital stays being responsible for an increase in resistance rates [21,22,52,55]. Indeed, previous studies on wild species [19,20,23,26,27], domestic animals [21,53,56], and humans [22,57] have correlated an increase in antimicrobial resistance to a hospitalization period. In our study, all individuals remained hospitalized for more than five days; thus, we did not observe shorter hospitalization periods. As such, our conclusions specifically apply to hospitalizations longer than five days. Nevertheless, we believe the study design adequately supports the role of hospitalization in AMR development. Between T0 and T1, the only other variable aside from hospitalization was antimicrobial treatment, which was explicitly included in the model to disentangle its effect from that of hospitalization itself.

Hypothetically, if animals with shorter hospital stays had been observed, two scenarios could have emerged: (1) no measurable increase in MIC values and AMR levels, which would suggest that a minimum duration of hospitalization is necessary to induce such changes; or (2) an observable increase even after a short hospitalization, indicating that even brief exposure to hospital conditions may suffice to promote AMR. This consideration reinforces the importance of minimizing the duration of hospitalization whenever possible and of implementing strict hygiene and biosecurity measures even during short stays.

Especially in WRCs, wild animals are exposed to a different diet (European hedgehogs are administered a mixture of pet food and insects [58]) that can alter the composition of the gut microbiota and promote its colonization by resistant strains [19,59]. Moreover, due to the frequent use of antimicrobials in hospital settings, susceptible bacteria from the environment can acquire or develop resistance genes and can persist over a period of up to eight years on hospital surfaces [60]. The use of disinfectants, like quaternary ammoniums, can also select for resistant bacteria (especially towards fluoroquinolones) [27,61]. In veterinary facilities and WRCs, animals come into close contact with hospital surfaces, like floors and cage walls, where AMR bacteria can be acquired [21,62]. Additionally, cages are usually located next to each other [63], promoting contact with other animals, and, while being fed and treated, animals also receive increased contact with humans [23,55,64]; both factors promote the acquisition of new, potentially resistant, bacteria [21]. Such exposures could have contributed to the selection of resistant bacterial subpopulations or may have promoted metabolic and structural changes in already present strains, such as increased expression of efflux pumps or altered cell wall permeability [2]. These findings should therefore be interpreted as a potential warning signal.

To reduce the potential acquisition and development of AMR bacteria in WRCs, responsible antibiotic use and the application of biosecurity measures are essential [23]. Proper hand hygiene and use of protective equipment, frequent disinfection, proper patient management and surveillance, personnel education, and a reduction in the duration of hospitalization could lead to reduced acquisition of AMR bacteria [55,61,63].

Sampling animals at WRCs provides a valuable opportunity to obtain biological material from wild individuals without the need for active capture in their natural environment. On the other hand, collected and hospitalized animals can suffer from acute or chronic diseases that could alter the composition of the intestinal microbiota [65], potentially promoting the proliferation of AMR bacteria and leading to their over-estimation. However, our detection rates were in line with previous studies performed on wild animals collected in their natural habitat, suggesting that this phenomenon, if it occurred, remained limited. An additional limitation is the absence of a marking system to identify individual hedgehogs. Although each animal was released in a different and safe area after recovery, we cannot fully exclude the possibility of readmissions of the same individuals. This could potentially lead to non-independence among some observations, and, in turn, to overestimating certain resistance patterns if repeated admissions occurred. Future studies should consider the use of non-invasive marking techniques to allow identification of recaptures and improve the robustness of results.

## 5. Conclusions

Our results confirm the relevant role of WRCs in the monitoring and study of wild species. We showed that, even in wild species, hospitalization for five days or more can favor the emergence of AMR, independently of antimicrobial administration. This underscores the importance of strict biosecurity and prudent antibiotic use in WRCs. Moreover, this study supports the role of European hedgehogs as carriers of phenotypically resistant *E. coli*, suggesting the possibility that they may contribute to the spread of AMR in the environment and to people who come into contact with them.

## Figures and Tables

**Table 1:** MIC distribution of the tested antibiotics at T0 and T1. The cells contain the frequency of isolates with a certain MIC among those assayed. Cells are shaded with a gradient of grey intensities based on the distribution of isolates: the darker the cell, the higher the frequency of isolates with a certain MIC. The table displays the median (MIC50) and the highest decile (MIC90) of MIC distribution, along with the (T)ECOFF value—where available—and the proportion of nWT *E. coli*. []: 95% CI of (T)ECOFF; (): 95% CI of nWT proportion.

		Tested MIC (µg/mL)														
Atb.	Time	0.12	0.25	0.5	1	2	4	8	16	32	64	128	MIC50	MIC90	(T)ECOFF	%nWT
AMI	T0						50	3	0	0	0		4	4	8 [4–8]	0.0 (0.0–0.0)
	T1						42	8	2	1	0		4	8		5.7 (0.0–11.9)
GEN	T0		0	13	21	16	0	1	2				1	2	2 [1,2]	5.7 (0.0–11.9)
	T1		1	7	15	23	3	0	4				2	4		13.2 (4.1–22.3)
AMP	T0		0	0	0	13	15	5	20				4	16	8 [4–16]	37.7 (24.7–50.8)
	T1		0	0	0	11	14	3	25				8	16		47.2 (33.7–60.6)
AMC	T0		0	0	0	9	17	16	11				8	16	(8) [2–64]	20.8 (9.8–31.7)
	T1		0	0	0	7	17	17	12				8	16		22.6 (11.4–33.9)
PIT	T0							53	0	0	0	0	8	8	8 [4–16]	0.0 (0.0–0.0)
	T1							50	3	0	0	0	8	8		5.7 (0.0–11.9)
CLE	T0			0	0	0	2	21	16	14			16	32	(32) [4–32]	†
	T1			0	0	0	0	18	16	19			16	32		†
CZO	T0				0	19	12	10	3	0	9		4	64	4 [0.5–16]	41.5 (28.2–54.8)
	T1				0	16	11	7	3	1	15		4	64		49.1 (35.6–62.5)
CPO	T0				43	6	2	0	2				1	2	2 [0.5–4]	7.5 (0.4–14.7)
	T1				41	4	4	0	4				1	4		15.1 (5.5–24.7)
CTZ	T0						53	0	0	0			4	4	1 [0.5–1]	†
	T1						51	1	0	1			4	4		†
CVE	T0		2	19	23	6	1	2	0				1	2	1	17.0 (6.9–27.1)
	T1		0	16	26	5	2	0	4				1	4		20.8 (9.8–31.7)
IMI	T0				53	0	0	0	0				1	1	0.5 [0.25–0.5]	†
	T1				53	0	0	0	0				1	1		†
CHL	T0					0	24	21	5	0	3		8	16	16 [8–16]	5.7 (0.0–11.9)
	T1					0	16	21	9	0	7		8	64		13.2 (4.1–22.3)
ENR	T0	41	5	1	0	0	0	6					0.12	8	0.125	22.6 (11.4–33.9)
	T1	31	6	4	0	0	0	12					0.12	8		41.5 (28.2–54.8)
MAR	T0	38	4	2	1	1	0	7					0.12	8	*	
	T1	32	3	4	1	0	1	12					0.12	8		
ORB	T0				40	4	1	1	7				1	16	*	
	T1				34	4	2	1	12				1	16		
PRA	T0		45	2	0	0	6						0.25	4	*	
	T1		36	5	0	0	12						0.25	4		
DOX	T0		0	0	22	21	3	3	4				2	8	4 [4–8]	13.2 (4.1–22.3)
	T1		0	0	6	29	3	4	11				2	16		28.3 (16.2–40.4)
TET	T0						46	0	0	7			4	32	8 [2–4]	13.2 (4.1–22.3)
	T1						39	0	0	14			4	32		26.4 (14.5–38.3)
TRS	T0			47	0	0	0	6					0.5	8	0.5 [0.125–1]	11.3 (2.8–19.9)
	T1			43	1	0	0	9					0.5	8		18.9 (8.3–29.4)

†: the proportion of nWT *E. coli* cannot be estimated because the (T)ECOFF fell outside the range of the tested MIC; *: no (T)ECOFF available.

## Data Availability

The raw data that were analyzed in this article are available upon direct request to the authors.

## Abbreviations

The following abbreviations are used in this manuscript:

Atb.	Antibiotic
AMC	Amoxicillin–clavulanic acid
AMI	Amikacin
AMP	Ampicillin
AMR	Antimicrobial resistance
BPW	Buffered peptone water
CBP	Clinical breakpoint
CHL	Chloramphenicol
CI	Confidence interval
CLE	Cefalexin
CPO	Cefpodoxime
CTZ	Ceftazidime
CVE	Cefovecin
CZO	Cefazolin
DOX	Doxycycline
ECOFF	Epidemiological cut-off
ENR	Enrofloxacin
ESBL	Extended-spectrum beta-lactamase
EUCAST	European committee on antimicrobial susceptibility testing
GEN	Gentamicin
IMI	Imipenem
MALDI-ToF	Matrix-assisted laser-desorption ionization—time of flight
McC3	McConkey 3 agar
MIC	Minimum inhibitory concentration
MIC50	50th percentile of the minimum inhibitory concentration
MIC90	90th percentile of the minimum inhibitory concentration
nWT	Non-wild-type
PIT	Piperacillin–tazobactam
T0	Time 0 (admission)
T1	Time 1 (discharge or approx. 10 days post admission)
TET	Tetracycline
TECOFF	Tentative epidemiological cut-off
TSB	Tryptone soya broth
TRS	Trimethoprim–sulfamethoxazole
WRC	Wildlife rescue center
WT	Wild-type
